# The most effective strategy for recruiting a pregnancy cohort: a tale of two cities

**DOI:** 10.1186/1471-2393-13-75

**Published:** 2013-03-22

**Authors:** Donna P Manca, Maeve O’Beirne, Teresa Lightbody, David W Johnston, Dayna-Lynn Dymianiw, Katarzyna Nastalska, Lubna Anis, Sarah Loehr, Anne Gilbert, Bonnie J Kaplan

**Affiliations:** 1 University of Alberta, Edmonton, AB, Canada; 2 University of Calgary, Calgary, AB, Canada

**Keywords:** Pregnant women, Research subjects, Cohort studies

## Abstract

**Background:**

Pregnant women were recruited into the Alberta Pregnancy Outcomes and Nutrition (APrON) study in two cities in Alberta, Calgary and Edmonton. In Calgary, a larger proportion of women obtain obstetrical care from family physicians than from obstetricians; otherwise the cities have similar characteristics. Despite similarities of the cities, the recruitment success was very different. The purpose of this paper is to describe recruitment strategies, determine which were most successful and discuss reasons for the different success rates between the two cities.

**Methods:**

Recruitment methods in both cities involved approaching pregnant women (< 27 weeks gestation) through the waiting rooms of physician offices, distributing posters and pamphlets, word of mouth, media, and the Internet.

**Results:**

Between May 2009 and November 2010, 1,200 participants were recruited, 86% (1,028/1,200) from Calgary and 14% (172/1,200) from Edmonton, two cities with similar demographics. The most effective strategy overall involved face-to-face recruitment through clinics in physician and ultrasound offices with access to a large volume of women in early pregnancy. This method was most economical when clinic staff received an honorarium to discuss the study with patients and forward contact information to the research team.

**Conclusion:**

Recruiting a pregnancy cohort face-to-face through physician offices was the most effective method in both cities and a new critically important finding is that employing this method is only feasible in large volume maternity clinics. The proportion of family physicians providing antenatal and post-natal care may impact recruitment success and should be studied further.

## Background

In recent years it has become more challenging to recruit pregnant women to volunteer for research and many strategies have been employed to increase participation. Studies have found that women decline to participate for a variety of reasons, including a growing number of requests to participate [1], time commitments [2,3], language barriers [2,4], the intrusiveness of the study (e.g., provision of biological specimens) [5,6], lack of interest and/or feeling that there is no perceived benefit from participation [5,7,8], and/or practical considerations such as holiday plans [9]. Although participants may be wary of committing to a project that requires a substantial amount of time, a review of large epidemiologic prospective pregnancy studies recruiting women prior to conception found that in some circumstances women are willing to be involved in time-consuming and/or invasive projects over an extended period of time [10].

Researchers have used a variety of methods to enlist pregnant women for studies. Approaching pregnant women directly in prenatal clinics or ultrasound clinics has resulted in response rates between 30% to 85% [5-7,11,12]. Many randomized clinical trials use media techniques, direct patient contacts, and contact with health care providers, with mixed success [13]. Media techniques that include an article describing the study published in a local newspaper have been effective for some [13]. Newspaper advertisements that do not have detailed study information or are run for a single day in a daily newspaper are less successful [13,14]. Pregnant women have also been successfully recruited via distribution of flyers and posters in locations they frequent such as hospitals [8,14], ultrasound clinics [8,14], laboratories [8,14], pre-natal classes [8], libraries [14], community centers [14], stores [14], coffee shops [14], trade shows and naturopathic and physiotherapy clinics [14].

The Alberta Pregnancy Outcomes and Nutrition project (APrON) is a longitudinal cohort study designed to examine the relationship between nutrient status during pregnancy, maternal mental health, and infant neurodevelopment and mental health. Participants were requested to attend assessments with the researchers once each trimester of their pregnancy and twice after delivery. The time commitment for each visit was approximately one to one and a half hours. They would continue to participate via mail out questionnaires on five occasions until their child was three years of age. The total time commitment was approximately 15 – 20 hours per participant over a three and a half year period. Details requirements from the study participants have been published elsewhere [15].

Health care in Canada is provided through a publically funded system. Medical services are governed by the Canada Health Act but are delivered provincially. The Canadian population does not pay for individual visits for medically necessary treatments. Maternity care is fully covered under the system. APrON recruited pregnant women in two cities, Calgary and Edmonton (Alberta). The cities were similar other than the arrangement of maternity care services. In Calgary, there are more high volume family physician-led clinics that provide maternity care. This is reflected in the proportion of births attended by a family physician. In Calgary in 2008, 37% of births were attended by a family physician while obstetricians attended 60% while in Edmonton, only 18% of births were attended by family physicians, while obstetricians attended 79% (Alberta Perinatal Health Program, 2010). Since women with obstetrician assisted births tend to receive their early prenatal care from family physicians, women in Edmonton were spread among many low volume family physicians’ offices as compared to the high volume family physician-led clinics in Calgary. This paper describes recruitment strategies used by APrON in these two cities, determines which were most successful and discusses reasons for the different success rates between the two cities.

## Methods

Ethics approval was obtained in 2009 from the Research Ethics Boards at the University of Alberta (HREB biomedical panel Pro00002954) and the University of Calgary (Conjoint Health Research Ethics Board ID E22101). Participants provided explicit consent prior to participation in the study.

Pregnant women were eligible to participate in APrON if they had a gestational age of less than 27 weeks on entry to the study. Gestation was determined through the women’s self-report of gestation and expected date of confinement and included consent for the researchers to access this information on the prenatal and deliver records. Women were ineligible if they were not fluent in English, or were planning to move out of the region in the next 6 months [15]. Numerous strategies were utilized to recruit the pregnancy cohort: some were employed in both cities, and others were unique to Edmonton.

### Recruitment strategies common to both cities

#### Physician Offices (family physicians, obstetricians and radiologists)

Family physician groups providing maternity care saw women early in their pregnancy and accepted referrals from physicians and self-referrals including repeat pregnancies. In Edmonton, in 2007, there were two main family physician groups providing prenatal care and attending women during labor and delivery. One group was estimated to attend 150 births each years and the other, 300 births each year. In Calgary, at the same time, there were three dedicated maternity clinics located at one location estimated to attend 2100 births each year.

A number of strategies involved face-to-face recruitment through physician offices. One method that was used in high volume maternity clinics (family medicine, obstetrical and radiology ultrasound) involved a receptionist directing pregnant women to a research assistant who was available in the waiting room to discuss the study. The research staff offered each pregnant woman a brochure briefly summarizing the project. In another method, clinic staff received a small honorarium or gift of appreciation to discuss the study with patients and forward contact information to the research team. This method was initially used in lower volume clinics but soon became the model of choice for both the low and high volume clinics due to economic feasibility.

#### Advertising

Posters and flyers were placed in public spaces that pregnant women were likely to frequent such as physicians’ clinics, hospital dietitians, laboratories, birth control centers, pharmacies, urgent care centres, ultrasound centres, grocery stores, maternity and baby stores, public libraries, zoos, botanical gardens, bookstores, yoga and fitness centers and toy stores.

#### Word of mouth

There was much effort expended in both cities to foster interest in the study via word of mouth from doctors, researchers and their staff, and friends and family of participants.

#### Media

APrON used earned-media (publicity gained through promotional efforts as opposed to paid media). We targeted media outlets with the greatest readership and audiences, as well as those related to pregnancy and nutrition. The study was highlighted on four television news stations (CBC, Global, CTV, and Shaw TV). Additionally, later in the recruitment period, an APrON advertisement ran for 24 hours on a smaller network (Access Network). Radio media was also used to promote the project (CBC French and English, CKUA, 660 News, and QR77). Print media included publications, press releases and stories in major newspapers, smaller papers, and magazines. Detailed information on the mass media including links to news articles are available on the APrON website at http://www.apronstudy.ca/Research/MassMedia.aspx#.

#### Internet

Recruitment solicitations were sent through various list servers such as emails through the Provincial Health Authority’s Primary Care Networks. Online recruitment involved the APrON website (http://www.apronstudy.ca/), and other postings such as University websites, social media included pages on Facebook (https://www.facebook.com/APrONstudy) and Twitter (https://twitter.com/apron_research), newspaper on-line articles and Mommy blogs. Participants were not recruited directly from Facebook or Twitter to avoid issues with exposing participants’ confidential information. The social medial pages posted information on APrON, including where and how to participate in the study.

#### Other

Recruiters were present at early pregnancy and nutrition classes 4 times a month (Calgary) and investigators presented at medical rounds in both cities. Study staff also contacted the Doula Association, Association for Safe Alternatives in Childbirth, midwives and naturopath clinics, centers for pregnant teens, programs to support low income pregnant women, community perinatal programs and the provincial after hours medical help line.

Community supporters such as child/maternity type stores and other businesses (see http://www.apronstudy.ca/GetInvolved/CurrentCommunitySupporters.aspx) who agreed to provide small gifts to participants were asked to spread the word and put the APrON link on their websites. These supporters also provided APrON with gifts that were used for a monthly APrON draw in exchange for free advertising with the supporters’ coupons and brochures. Recruiters also attended events such as baby day events in major shopping retailers and malls, craft shows, and a variety of baby fairs, pregnancy trade shows, Welcome Wagon baby showers, and other celebrations and festivals.

### Recruitment unique to Edmonton

There were some important differences between the two cities in strategies used. In Edmonton, APrON initially recruited primarily through the Women and Children’s Health Research Institute (WCHRI), which had established a network to recruit pregnant women to all academic pregnancy studies conducted in that city. In addition to APrON, WCHRI was simultaneously recruiting for three other studies.

The WCHRI mode of recruitment involved asking obstetricians and family physicians caring for a high volume of pregnant women to invite their patients to learn more about the study by permitting their contact information to be faxed to the WCHRI central office. WCHRI then took responsibility for forwarding patients’ contact information to the appropriate study. If a woman was eligible for more than one study, she was randomly assigned to one of the active studies. Upon receiving the potential participant’s contact information, APrON would contact the woman to describe the study, review eligibility criteria, and arrange the first on-site visit. Each group practice with a minimum of 3 physicians was remunerated at $250/month by WCHRI.

### Considerations other than recruitment strategies

Other factors also affected recruitment. The APrON research sites were initially located in NorthWest Calgary at the Alberta Children's Hospital and in Edmonton on the University Campus. The populations in these areas are of a higher socioeconomic status (SES) and more highly educated than some other areas of the cities. Participants were required to attend these centres to complete surveys and other measures. In order to increase the ethnic diversity of the sample, multiple satellite clinics were later opened in areas of Calgary with a more diverse population, and closer to public transit.

## Results

Recruitment activities such as advertising were initiated in January 2009 and APrON participant recruitment began in May 2009. Of the first 1,200 women recruited, 1,028 (86%) were in Calgary while only 172 (14%) were in Edmonton. In 2008 there were 50,164 live births in Alberta with 18,633 live births occurring in Calgary and 14,866 in Edmonton respectively [16]. Thus based upon the proportion of live births alone, we could expect to recruit 56% (18,633/33,399) of our sample from Calgary and 44% (14,866/33,399) from Edmonton.

In the first year of the study, many of the women were not asked how and where they heard about the APrON project. Of those who were subsequently asked, Table 1 illustrates the number of participants recruited in Edmonton and Calgary through all recruitment strategies as reported by the women.

**Table 1:** Recruitment strategies as reported by the women in Edmonton and Calgary

**City**	**Edmonton**	**Calgary**
*Strategy*	*Number (%)*	*Number (%)*
	*172/1200 (14)*	*1028/1200 (86)*
Recruitment in medical offices by physicians and office staff	35 (22.0)	276 (33.6)
Recruitment at medical/ultrasound clinics by APrON staff	4 (2.5)	180 (21.9)
Poster/Pamphlet in medical offices, ultrasound clinics, play group etc.	19 (12.0)	108 (13.2)
Word of mouth from participants; family or friends who are not participants, or from a university employee	37 (23.3)	48 (5.9)
Media – newspaper, magazine	12 (7.5)	7 (0.9)
Media – TV, radio	20 (12.6)	19 (2.3)
Internet – list servers (Health Notes) and others	20 (12.6)	1 (0.1)
Internet – APrON website	9 (5.7)	12 (1.5)
Other	3 (1.9)	170 (20.7)*
Total	159 (100)	821 (100)

Women reported on the one method they identified as responsible for recruiting them into the APrON project.

*Other for Calgary includes the following: Pregnancy/Nutrition Class: 23 (2.8); Baby Fairs: 13 (1.6).

Media Public Service Announcement (unpaid media): 5 (0.6); Other: 115 (14.0).

### Edmonton

The WCHRI-based recruitment that included multiple studies was notably unsuccessful for APrON. APrON only received 107 referrals from WCHRI and of those, only 19 (18%) joined the Edmonton APrON cohort. A decision was made to cease recruitment through the WCHRI strategy in September 2010 and active recruitment continued outside the WCHRI mode until November 2010 when a sample of 1,200 was complete.

During the first two weeks at the family practice clinic selected for trialing on-site recruitment in Edmonton, the APrON research assistant spoke to 44 pregnant women. Of these, 15 (34%) were over 27 weeks gestation; seven declined for other reasons including being too busy (N=1), being uncomfortable with the blood samples (N=2), or no reason indicated (N=4); five took the information but did not want a follow-up call or email; one was already involved with APrON; 13 (30%) provided their contact information (email and/or phone number) for follow up and three (7%) booked appointments directly for the research project. After the first two weeks, the majority of women coming to the clinic had already spoken to the researchers.

Additional strategies employed in Edmonton are described in Table 1. Of the 159 Edmonton women who reported on recruitment strategies, the most successful strategies were face-to-face conversations in physician and ultrasound offices (39), word of mouth (37), TV media coverage (20) and advertising through posters and pamphlets (19). Of note, a higher proportion of Edmonton women were recruited by word of mouth than in Calgary. We think this difference was likely due to the inability to obtain adequate access to pregnant women in the clinical setting; hence word of mouth played a larger role in recruitment in Edmonton.

### Calgary

As is clear from Table 1, face-to-face recruitment was also the most effective strategy in Calgary: of 821 Calgary participants, 276 women were obtained from recruiters in doctors’ offices, and 180 from recruiters in ultrasound offices.

These results were consistent with those observed in the subsample of 196 women who participated via the satellite clinics established for the purpose of diversifying the Calgary sample. In that subgroup, the most successful strategy was to fully educate the family doctors, who then presented the study to those patients who were potentially interested. As most of the women with diverse cultural backgrounds and/or low SES sought care mostly, if not exclusively, through their family doctor, this approach allowed us the opportunity to access these women the best. The final result was that 62% (122/196) of these women were recruited after their family doctor first introduced the study and asked if they would be willing to hear more from a research assistant, 14% (27/196) were first approached by a research assistant, and 24% (47/196) were recruited by a handful of the other recruitment strategies.

### Comparison of sociodemographic variables

Maternal age, income, education, employment status and ethnicity were similar in both cities (Table 2) and though Calgary had a slightly higher SES there were no significant differences in the data using non-parametric statistical analysis (Fisher’s exact test and Chi-square). However, Calgary was able to recruit a small number of participants without high school education whereas all of Edmonton’s participants had high school education or higher.

**Table 2:** **Description of Calgary and Edmonton APrON Participants**^**1**^

**Calgary**			**Edmonton**			**Total**		
	**N**	**Percentage within site**	**Percentage of total**	**N**	**Percentage within site**	**Percentage of total**	**N**	**Percentage of total**
**Income**								
< $20,000	21	2.1%	1.8%	0	0.0%	0.0%	21	1.8%
$20,000-$39,000	38	3.9%	3.3%	9	5.4%	0.8%	47	4.1%
$40,000-$69,000	130	13.3%	11.3%	18	10.7%	1.6%	148	12.9%
$70,000-$99,00	209	21.3%	18.2%	49	29.2%	4.3%	258	22.5%
$100,000+	581	59.3%	50.7%	92	54.8%	8.0%	673	58.7%
Total	979 (missing 49)			168 (missing 4)			1147 (missing 53)	
**Education**								
Trade/	893	90.2%	77.0%	154	91.1%	13.3%	1047	90.3%
Under-graduate/								
Post Graduate								
High School/<	97	9.8%	8.4%	15	8.9%	1.3%	112	9.7%
High School								
Total	990 (missing 38)			169 (missing 3)			1159 (missing 41)	
**Working at a paid job**								
Yes	732	72.6%	62.1%	131	76.6%	11.1%	863	73.2%
No	276	27.4%	23.4%	40	23.4%	3.4%	316	26.8%
Total	1008 (missing 20)			171 (missing 1)			1179 (missing 21)	
**Marital status**								
Married/	950	96.3%	82.0%	165	96.5%	14.2%	1115	96.3%
Common-Law								
Single/	37	3.7%	3.2%	6	3.5%	0.5%	43	3.7%
Divorced/								
Separated								
Total	987 (missing 41)			171 (missing 1)			1158 (missing 42)	
**Ethnicity**								
Caucasian	841	86.3%	73.6%	146	86.9%	12.8%	987	86.4%
Non-Caucasian	134	13.7%	11.7%	22	13.1%	1.9%	156	13.6%
Total	975 (missing 53)			168 (missing 4)			1143 (missing 57)	

^1^Denominators vary due to missing data.

## Discussion

The most effective strategy overall was face-to-face recruiting through physicians’ offices, including ultrasound offices. The cost of face-to-face recruiting is highly influenced by the structure of the health care delivery model. In Edmonton, early pregnancy care is distributed throughout the city in family physician offices and later care in obstetrician offices, whereas in Calgary about half of the early pregnancy care occurs in high volume family medicine obstetrical clinics. This difference likely affected APrON’s recruitment success. The distribution of pregnant women in many clinics in Edmonton was a challenge as we could not afford the cost of establishing so many recruitment sites: there are more than 1000 Edmonton family physicians registered with the Alberta College of Physicians and Surgeons. Others have described access to eligible women as a limitation of recruiting pregnant women directly in clinical settings [6,11,14]. Also, having more prenatal care provided by obstetricians meant that it was more difficult to recruit women < 27 weeks gestation. Although placing recruiters in physician offices was more cost effective in the high volume family medicine maternity clinics in Calgary, it was still more efficient to pay an honorarium to the respective practice nurses. Hence a city that has a higher proportion of family physicians providing antenatal care and attending women during labor and delivery may enhance the success of researchers attempting to access pregnant women. Recruiting from waiting rooms may have been more effective if there had been no restriction on gestational age.

In Edmonton, with the lack of access to high volume early maternity clinics, the media appeared to be particularly successful in gaining interest in the APrON study. It is important to note that these were actual media stories about the research project rather than paid advertisements seeking recruits. Other research has confirmed that media coverage is more successful than media advertisements in recruiting women to studies [13]. Posters and pamphlets in medical offices were also productive, which is consistent with the report of other research where flyers/posters garnered the greatest interest in their pregnancy related study [14].

Although not calculated precisely, research assistants reported that many women recruited in physicians’ offices had already heard about APrON. Media coverage and other advertisement exposure may prime potential participants to be receptive to enrolling in a research project, so that even though their recruitment is attributed to direct approach in physicians’ offices, the media heightening awareness of the study was likely influential.

There were, however other variations in recruitment methods that may have contributed to the differences in success between the two cities. In Edmonton a collaborative method was used through WCHRI, which may not have been as successful in providing access to eligible women because APrON was competing with other projects and the women did not have a choice as to which project they were assigned. Recruitment through WCHRI primarily involved obstetricians which also may account for the poor success in accessing women who were < 27 weeks gestation.

Another difference between Edmonton and Calgary was the variation in the scheduling of women attending radiology clinics for first and second trimester fetal screening. In Calgary, researchers were able to access numerous pregnant women who attended the ultrasound clinic via block scheduling while in Edmonton pregnant women were mixed in with other patients being seen for health concerns, limiting the number of pregnant women in the clinic at any one time.

On average, the participants from both cities were of relatively high SES. In Calgary, the high SES of the participants may have been influenced by the location of the primary APrON research site. This unit is difficult to access by transit and thus participants needed a car. On the other hand, other researchers have found that pregnant women of higher SES are generally more likely to participate in pregnancy-related research studies, so perhaps the skewed SES would have occurred no matter where the research sites were located [8,14]. APrON participants were also more highly educated than the general population with almost all of them having obtained at least high school education, that is, only 9.7% had only high school or less than a high school education. In the total Alberta population of women aged 25 to 34 in 2005, 12% (27,845/234,175) did not have a high school education (no certificate, diploma or degree) [17]. The higher educational level of volunteers is consistent with the findings of other studies. In one cohort study of 152 pregnant women [14], women were somewhat older, predominantly Caucasian, more affluent, and better educated than the general population of pregnant and non-pregnant women residing in Vancouver [14], and in an Italian internet-based mother-child cohort study, participants were older, had higher educational levels, and were more often primiparous [8]. Although in general the two Alberta cities are quite similar, Calgary’s population appears to have a slightly higher SES than Edmonton’s with a higher overall median family income (77,658 versus 69,214), fewer individuals ≥ 15 years of age without certificates or diplomas 18% (145,125/801,265) versus 22% (131,220/598.905), and a lower unemployment rate of 4.1 versus 4.9 [17,18]. While Edmonton’s population is slightly smaller and consists of a slightly lower SES [17] it seems unlikely that this difference alone would explain the large disparity in recruitment success between the two cities. It is more likely that the greater success in Calgary was attributable to the availability of family physician-led high volume maternity clinics.

## Conclusion

In summary, we had the unusual opportunity of being able to compare recruitment strategies and success in two very similar cities in a single study. It is an interesting finding that in both cities the most successful method was through face-to-face strategies in physician offices, a method that is feasible only in settings that have access to large volume maternity clinics with women in early pregnancy, a new critically important finding. Hence, we concluded that a major influence on recruitment success was the health care model, which differed between the two cities. Our results suggest that the proportion of family physicians providing antenatal and post-natal care may be a significant factor in determining recruitment success and should be studied further.

## Competing interests

None of the authors have any personal or financial conflict of interest.

## Authors’ contributions

DM – involved in development of the recruitment strategy and follow-up, conceived and designed the paper, involved with conducting the initial literature review, wrote the first draft manuscript. MO – involved in development of the recruitment strategy and follow-up, conceived and designed the paper, reviewed the initial literature review. TL - involved in development of the recruitment strategy and follow-up, conceived and designed the paper, involved with conducting the initial literature review, wrote the first draft manuscript. DJ - involved in development of the recruitment strategy and follow-up, helped to conceive and design the paper, involved with reviewing the initial literature review, assisted with the drafts. DD - involved in development of the recruitment strategy and follow-up, contributed to the conception, composition of the article. KN- involved in implementation of the recruitment strategy and follow-up, contributed to the conception, composition of the article. LA - involved in development of the recruitment strategy and follow-up, contributed to the conception, composition of the article. SL - contributed to the conception, composition of the article. AG - contributed to the conception, composition of the article. BK – principal investigator, involved in development of the recruitment strategy and follow-up, conceived and designed the paper, reviewed the initial literature review. All authors were involved in the drafts and revisions and read and approved the final manuscript.

## Pre-publication history

The pre-publication history for this paper can be accessed here:

http://www.biomedcentral.com/1471-2393/13/75/prepub
